# Cas-OPRAD: a one-pot RPA/PCR CRISPR/Cas12 assay for on-site *Phytophthora* root rot detection

**DOI:** 10.3389/fmicb.2024.1390422

**Published:** 2024-06-05

**Authors:** Zhiting Li, Wanzhen Feng, Zaobing Zhu, Shengdan Lu, Mingze Lin, Jiali Dong, Zhixin Wang, Fuxiu Liu, Qinghe Chen

**Affiliations:** ^1^School of Breeding and Multiplication, School of Tropical Agriculture and Forestry, Hainan University, Sanya, China; ^2^Key Laboratory of Green Prevention and Control of Tropical Plant Diseases and Pests, Ministry of Education, Hainan University, Haikou, China; ^3^Institute of Plant Protection, Jiangsu Academy of Agricultural Sciences, Nanjing, China; ^4^Sanya Institute of China Agricultural University, Sanya, China; ^5^Post-Entry Quarantine Center for Tropical Plant, Haikou, China

**Keywords:** *Phytophthora sojae*, one-pot RPA/PCR, recombinase polymerase amplification, CRISPR/Cas12a, on-site detection, visual detection

## Abstract

*Phytophthora sojae* is a devastating plant pathogen that causes soybean *Phytophthora* root rot worldwide. Early on-site and accurate detection of the causal pathogen is critical for successful management. In this study, we have developed a novel and specific one-pot RPA/PCR-CRISPR/Cas12 assay for on-site detection (Cas-OPRAD) of *Phytophthora* root rot (*P. sojae*). Compared to the traditional RPA/PCR detection methods, the Cas-OPRAD assay has significant detection performance. The Cas-OPRAD platform has excellent specificity to distinguish 33 *P. sojae* from closely related oomycetes or fungal species. The PCR-Cas12a assay had a consistent detection limit of 100 pg. μL^−1^, while the RPA-Cas12a assay achieved a detection limit of 10 pg. μL^−1^. Furthermore, the Cas-OPRAD assay was equipped with a lateral flow assay for on-site diagnosis and enabled the visual detection of *P. sojae* on the infected field soybean samples. This assay provides a simple, efficient, rapid (<1 h), and visual detection platform for diagnosing *Phytophthora* root rot based on the one-pot CRISPR/Cas12a assay. Our work provides important methods for early and accurate on-site detection of *Phytophthora* root rot in the field or customs fields.

## 1 Introduction

*Phytophthora sojae* is a major oomycete pathogen that causes damping-off of seedlings, and root and stem rot of soybean plants in soybean-producing regions worldwide (Erwin and Ribeiro, 1996; Tyler, 2007). *Phytophthora sojae* can infect soybean at all growth stages and yield losses can reach 100% in soybean fields when susceptible varieties are planted (Wang et al., 2006; Dorrance, 2018). *Phytophthora sojae* is listed as a plant quarantine pathogen in China due to its high risk to agricultural and economic security (Wang et al., 2006; Dai Y. et al., 2019). With the increasing global transport of soybeans, *P. sojae* presents a serious threat to soybean production worldwide. *Phytophthora* root rot is difficult to diagnose due to the symptoms resembling those caused by other pathogens such as *Fusarium*, *Pythium*, or *Rhizoctonia* species. Therefore, the development of a new technique for the detection of the pathogen will be essential to prevent transmission of *P. sojae* into disease-free soybean growing regions.

Various methods are available for detecting *P. sojae* infection, isolation and cultivation, leaf disc baiting, and molecular detection. Isolation-based methods offer incontrovertible evidence of *P. sojae* infection (Su and Shen, 1993; Chen et al., 2004), while the leaf-disc baiting assay is less complicated and more effective than isolating the pathogen from plant tissues (Jinhuo and Anderson, 1998; Dai T. et al., 2019; Matthiesen et al., 2021). Molecular approaches, such as conventional polymerase chain reaction (PCR) and real-time PCR (qPCR), are well-established and considered powerful diagnosis tools (Bienapfl et al., 2011; Haudenshield et al., 2017; Xiong et al., 2019). However, culturing and baiting detection methods are time-consuming and relatively insensitive. Furthermore, conventional PCR or real-time PCR require well-equipped laboratories and well-trained personnel, hindering their application in resource-limited environments or on-site detection. In contrast, various isothermal nucleic acid amplification techniques, such as loop-mediated isothermal amplification (LAMP) and recombinase polymerase amplification (RPA), allow nucleic acid amplification at a single temperature, making them suitable for field and on-site diagnosis in low-resource areas (Dai et al., 2012; Rojas et al., 2017). However, their practical application remains challenging due to the issues of false positives caused by non-specific amplification.

The clustered regularly interspaced short palindromic repeat (CRISPR) and CRISPR-associated (Cas) system has recently been applied for molecular detection (Gootenberg et al., 2017; Chen et al., 2018; Li et al., 2018; Wheatley and Yang, 2021; Eisenstein, 2022). Several CRISPR/Cas-based detection platforms, such as DETECTR (Chen et al., 2018), HOLMES (Li et al., 2018), and SHERLOCK (Gootenberg et al., 2017; Kellner et al., 2019), have been developed as point-of-care diagnostic tools for highly specific and sensitive detection of the pathogen. CRISPR-based molecular detection offers several advantages, including rapid detection times, high specificity, and sensitivity comparable to or exceeding traditional methods like PCR (Islam and Kasfy, 2023; Kasfy et al., 2024). Additionally, these methods are relatively simple and can be adapted for use in various settings, including point-of-care diagnostics and field surveillance (Li et al., 2019; Islam and Kasfy, 2023). Most recently, Cas12a-based detection methods have been used to diagnose plant pathogenic fungi (Magnaporthe oryzae), bacterial pathogens (Candidatus Liberibacter asiaticus), and viruses (PVX, PVY, TMV, ASPV, RSV) (Aman et al., 2020; Jiao et al., 2021; Kang et al., 2021; Wheatley and Yang, 2021; Sánchez et al., 2022; Zhu et al., 2022). However, these platforms currently employ step-by-step nucleic acid preamplification and Cas12a cleavage steps, which could potentially increase the risk of carryover contamination between processes. This is particularly concerning for specific detection, as the extra processes of tube opening during preamplification can introduce non-specific amplification and cross-contamination (Wang SY et al., 2021; Guo et al., 2023). To address these challenges, there is a need to develop one-pot CRISPR/Cas-based assays for plant pathogen detection. Such assays would integrate the preamplification and cleavage steps into a single reaction, reducing the risk of contamination and improving specificity.

In this study, we present a novel one-pot RPA/PCR CRISPR/Cas12a assay for the simple, rapid, specific, and on-site visual detection of *P. sojae*, combining either PCR or RPA with CRISPR/Cas12a. Following amplification, an optimized Cas12a solution was added to the reaction mixture to initiate cleavage. The results were visualized by naked-eye observation under blue or UV light. To facilitate in-field detection in resource-limited settings, we further combined the CRISPR/Cas12a assay with lateral flow strips. Except for the analytical sensitivity and specificity, the feasibility of this CRISPR/Cas12a assay was evaluated using inoculated soybeans samples and field samples. The Cas-OPRAD assay is a simple, specific, sensitive, and visual method for *P. sojae* detection, which has the potential to be widely applied in the management of plant pathogens.

## 2 Materials and methods

### 2.1 Isolates and DNA extraction

A total of 33 *P. sojae* isolates from different geographic areas were tested in this study, as well as 37 isolates from 17 different oomycetes (including 34 isolates of 14 species representing 10 clades of *Phytophthora*), and 16 other fungi and bacteria isolated from soybean and other hosts. The origin, host affiliation, and number of tested isolates are listed in Table 1. All isolates were routinely maintained in liquid nitrogen at the School of Breeding and Multiplication, Hainan University, China.

**Table 1:** Isolates pathogens used in this study.

	Species^a^	Host	Number of isolates	Source	Result^b^	
					PCR-Cas2a	RPA-Cas12a
1	*Phytophthora sojae*	*Glycine max*	8	Fujian, China	+	+
2	*Phytophthora sojae*	*Glycine max*	9	Hainan, China	+	+
3	*Phytophthora sojae*	*Glycine max*	4	Heilongjiang, China	+	+
4	*Phytophthora sojae*	*Glycine max*	5	Anhui, China	+	+
5	*Phytophthora sojae*	*Glycine max*	7	Zhangzhou, China	+	+
6	*Phytophthora infestans*	*Solanum tuberosum*	3	Fujian, China	_	_
7	*Phytophthora vignae*	*Vigna unguiculata*	4	Hainan, China	_	_
8	*Phytophthora colocasiae*	*Colcasia esculenta*	5	Hainan, China	_	_
9	*Phytophthora nicotianae*	*Nicotiana tabacum*	2	Fujian, China	_	_
10	*Phytophthora capsici*	*Capsicum annuum*	5	Hainan, China	_	_
11	*Phytophthora cactorum*	*Malus pumila*	2	Jiangsu, China	_	_
12	*Phytophthora parasitica*	*Ananas comosus*	3	Hainan, China	_	_
13	*Phytophthora drechsleri*	*Beta vulgaris*	1	Fujian, China	_	_
14	*Phytophthora boehmeriae*	*Gossypium*	1	Jiangsu, China	_	_
15	*Phytophthora citrophthora*	*Citrus reticulata*	1	Fujian, China	_	_
16	*Phytophthora melonis*	*Cucumis meloa*	2	Fujian, China	_	_
17	*Phytophthora palmivora*	*Dracaena sanderiana*	3	Hainan, China	_	_
18	*Phytophthora cinnamomi*	*Persea americana*	1	Hainan, China	_	_
19	*Phytophthora cryptogea*	*Gerbera jamesonii*	1	Fujian, China	_	_
20	*Peronophthora litchi*	*Litchi chinensis*	1	Fujian, China	_	_
21	*Pythium aphanidermatum*	*Cucumis sativus*	1	Fujian, China	_	_
22	*Phytopythium* spp.	*Citrus reticulata*	1	Jiangsu, China	_	_
23	*Fusarium solani*	*Glycine max*	1	Hainan, China	_	_
24	*Sclerotinia sclerotiorum*	*Glycine max*	1	Hainan, China	_	_
25	*Colletotrichum gloeosporioides*	*Glycine max*	1	Hainan, China	_	_
26	*Alternaria alternata*	*Glycine max*	1	Fujian, China	_	_
27	*Rizoctonia solani*	*Glycine max*	1	Fujian, China	_	_
28	*Phomopsis longicolla*	*Glycine max*	1	Jiangsu, China	_	_
29	*Verticillium dahliae*	*Solanum melongena*	1	Fujian, China	_	_
30	*Fusarium oxysporum*	*Gossypium hirsutum*	1	Fujian, China	_	_
31	*Fusarium moniliforme*	*Gossypium hirsutum*	1	Fujian, China	_	_
32	*Fusarium graminearum*	*Triticum aestivum*	1	Fujian, China	_	_
33	*Botryosphaeria rhodina*	*Psidium guajava*	1	Fujian, China	_	_
34	*Helminthosprium turcicum*	*Zea mays*	1	Fujian, China	_	_
35	*Magnaporche oryzae*	*Oryza sative*	1	Fujian, China	_	_
36	*Colletotrichum siamense*	*Hevea brasiliensis*	1	Hainan, China	_	_
37	*Erwinioca rotovora*	*Solanum tuberosum*	1	Fujian, China	_	_
38	*Ralstonia solanacearum*	*Solanum tuberosum*	1	Fujian, China	_	_

^a^All isolates were maintained in the collection of Hainan University. ^b^Positive (+) or Negative (−) are based on the presence of a PCR-Cas12a or RPA-Cas12a result of amplification.

Genomic DNA was extracted from mycelial cultures using a DNAsecure Plant kit (Tiangen, Beijing, China) as described previously (Wu et al., 2017). The purified genomic DNA was quantified using a NanoDrop 2000 spectrophotometer (Thermo Scientific), and stored as aliquots at a concentration of 100 ng μL^−1^ in sterile distilled water at −20°C.

### 2.2 Materials and reagents

All the primers, DNA fragments (including CRISPR RNA [crRNA], and ssDNA probe) were synthesized by Sangon Biotech Co., Ltd. (Shanghai, China). REnGen® Lba Cas12a and NEBufferTM were purchased from New England BioLabs Ltd. (Beijing, China). High-Affinity HotStart Taq (ET108-1) and 2 × PCR SuperMix were purchased from Tiangen Biotech (Beijing, China), while RPA Kits were obtained from AMP-Future Biothech Co., Ltd. (Changzhou, China). The lateral flow strips were purchased from Tiosbio Biotech Co. Ltd. (Beijing China). Nuclease-free H_2_O was obtained from Sangon Biotech (Shanghai, China). Genomic DNA Kits were purchased from Tiangen Biotech (Beijing, China).

### 2.3 Comparative genomic analysis and detection target

To identify the conserved, single-copy, candidate detection targets for *P. sojae* specific PCR or RPA assays, we retrieved the annotated genomic sequence of *P. sojae*.^1^ All the gene sequences of *P. sojae* were used to perform BLAST searches against the publicly available genomic sequences of *Phytophthora* and related taxa (e.g., *Pythium*, and *Phytopythium* species) in the National Center for Biotechnology Information (NCBI) database. A total of 1,187 *P. sojae* genes did not share any sequence similarity with genomic sequences of any other species. Three loci (PHYSODRAFT_299276, PHYSODRAFT_255386, and PHYSODRAFT_531894) were randomly selected as detection targets to test the specificity using a collection of *Phytophthora* species (Table 1) in conventional PCR. Ultimately, a single-copy gene (PHYSODRAFT_299276) was selected as the specific target for *P. sojae* detection.

### 2.4 Primers and CRISPR RNA design

The target sequence of *P. sojae* (PHYSODRAFT_299276) was used to design the crRNAs and primers for the CRISPR/Cas assay. RPA primers were designed and analyzed using Primer 5 and Primer-Explore V5 software.^2^ The crRNA with 20–24 bp target-dependent sequence following PAM (5’-TTTN-3′) sit was designed using EuPaGDT^3^ and an RNA scaffold to assist in protein binding. RPA primers and crRNA were synthesized by Sangon Biotech (Shanghai, China). The sequences of all primers are listed in Supplementary Table S1.

### 2.5 Conventional PCR assay

Conventional PCR assays were performed in 10 μL reactions. Each reaction included 5 μL of 2 × Taq PCR Master Mix (Tiangen Biotech Co., Ltd., Beijing, China), 0.5 μL of each forward and reverse primer (10 μM), 1 μL of template DNA (100 ng μL^−1^), and 3.0 μL of nuclease-free H_2_O. Thermal cycling was carried out using PTC-200 Thermo Cycler (MJ Research, Watertown, MA, United States) with the following conditions: 95°C for 5 min, followed by 30 cycles of 95°C for 30 s, 58°C for 45 s, and 72°C for 30 s, and a final extension at 72°C for 10 min. The PCR amplification products were separated by electrophoresis on 2.0% agarose gel, stained with ethidium bromide, and visualized under UV light. Each experiment was performed in triplicate.

### 2.6 Recombinase polymerase amplification assay

RPA assay was performed using the AMP-Future Biotech Co. Ltd. (Changzhou, China) according to the manufacturer’s instructions. Briefly, each 10 μL reaction contained 5.9 μL of A buffer, 0.5 μL of B buffer, 0.4 μL of each forward and reverse primers (10 μM), 1 μL of DNA (100 ng μL^−1^), and 0.8 μL of nuclease-free H_2_O. The mixture was incubated in a conventional water bath at 38°C for 15 min for RPA reaction.

### 2.7 Optimization of CRISPR/Cas12a detection

The efficiency of the CRISPR/Cas12a cleavage system is directly influenced by the concentration of crRNA and Cas12a and affects both trans-cleavage efficiency and fluorescence intensity. To determine the optimal CRISPR/Cas12a reaction conditions, a range of Cas12a and crRNA concentrations (50 nM ~ 300 nM), Cas12a-mediated cleavage temperature (37 ~ 42°C), RPA reaction time (5 min ~ 30 min), and trehalose concentrations (0.006 mM ~ 6 mM) were tested. Nuclease-free water was used as a negative control. The CRISPR/Cas12a reaction products were analyzed by naked eye observation or by detecting the maximal fluorescence signal value to determine the optimal CRISPR/Cas reaction conditions. All experiments were repeated three times.

### 2.8 CRISPR/Cas12a-mediated cleavage assay

The CRISPR/Cas12a reaction was performed in a 5 μL reaction mixture containing 0.75 μL crRNA (4 μM), 1.5 μL Cas12a (2 μM), 0.75 μL ssDNA FQ reporter (100 nM), and 1.5 μL of 10× NEB Buffer r2.1, 0.5 μL of nuclease-free H_2_O, along with 10 μL of RPA products (or 0.5 μL of 6 mM trehalose and 10 μL of PCR products). In the PCR-CRISPR/Cas12a assay, 6 mM trehalose was added to the reaction solution to protect the activity of the Cas12a protein. The reactions were incubated at 38°C for 10 min and then placed on an ultraviolet (UV) or blue light transilluminator for naked-eye detection.

### 2.9 One-pot CRISPR/Cas12a assay

**Figure 1:**
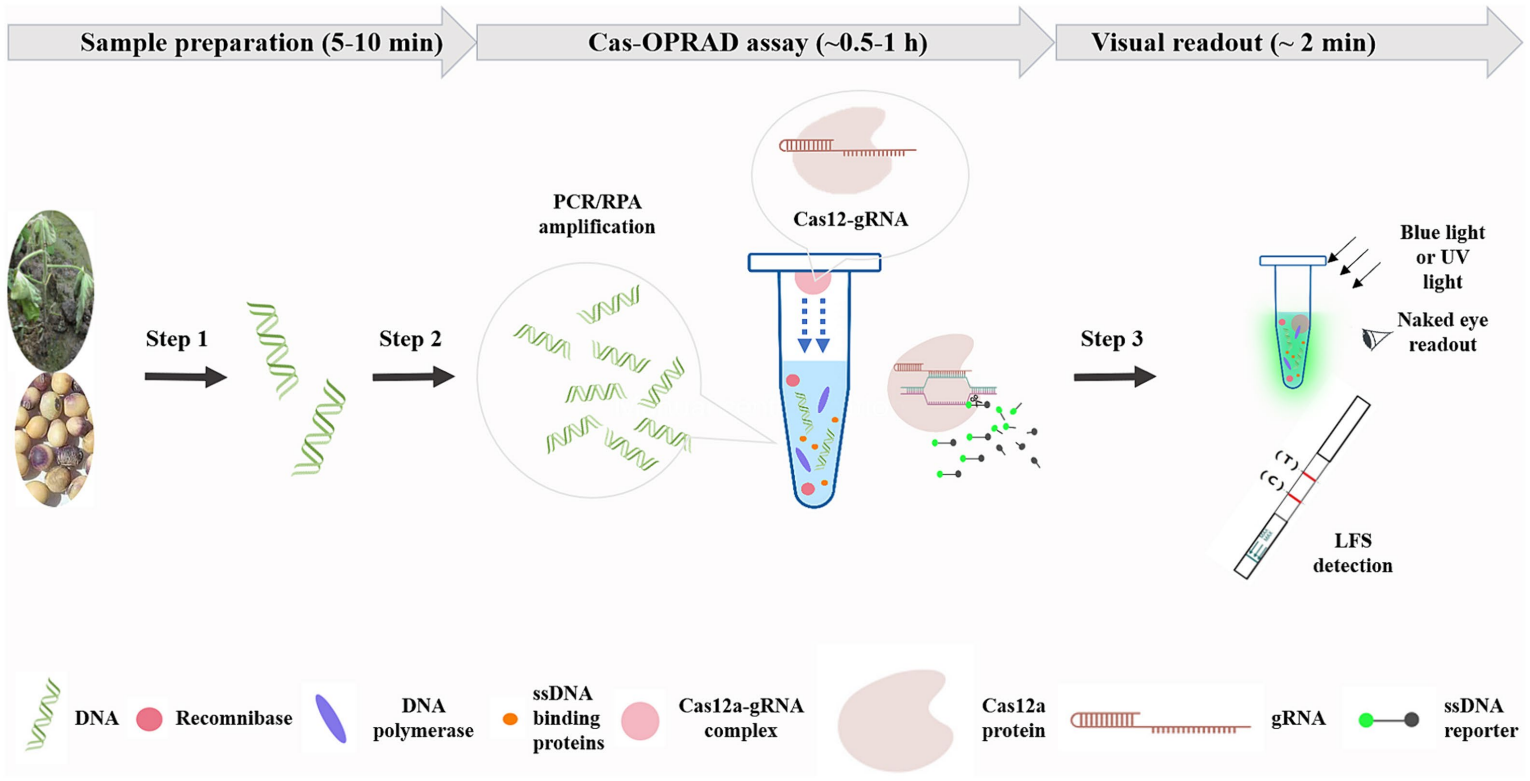
Schematic of Cas-OPRAD workflow for rapid sensitivity, and visual detection of *P. sojae*. Step 1: sample preparation. Step 2: Cas-OPRAD assay. Step 3: visual readout.

The one-pot CRISPR/Cas12a detection combines RPA/PCR reaction with Cas12a cleavage in a single tube reaction. To perform the assay, 10 μL of the RPA/PCR assay containing targets was added to the bottom of the tube, and 5 μL of CRISPR/Cas12a reaction solution was initially added inside the inner wall of the tube lid. After incubating the RPA/PCR reaction for 10–60 min, the CRISPR/Cas12a solution was swung into the RPA reaction solution by hand on-site, and the mixture was incubated for an additional 15 min. The reaction tubes were then placed on a blue or UV light transilluminator for naked eye detection (Figure 1).

### 2.10 Lateral flow assay

The structure of the lateral flow strip (LFS) incorporated an absorbent pad, a conjugate pad, a nitrocellulose (NC) membrane, and a sample pad on a plastic adhesive backing card. The capture reagents streptavidin and anti-IgG antibody were dispensed onto the NC membrane, and each band was separated by 5 mm. When there is no target in the system, the conjugate was captured by the streptavidin through biotin, the control line became red because of the aggregation of the colloidal gold in this area, while the test line without the flow-through of the conjugate had no color change. However, if there are targets in the system, the ssDNA reporter is cleavaged by CRISPR/Cas12a, and FAM and biotin molecules are separated. After the integration of ssDNA-biotin captured by streptavidin at the C line, the remaining conjugate of FAM-ssDNA and anti-FAM antibody would flow into the test line and be captured by anti-lgG antibody. The lateral flow detection was performed according to the Tiosbio protocol (Tiosbio Biotech Co. Ltd., Beijing China). Briefly, 85 μL Tiosbio assay buffer was added to 15 μL of the RPA-CRISPR/Cas12a reaction and incubated for 5 min. The lateral flow test strip was then inserted into the reaction mixture for 5 ~ 10 min to allow the reaction to occur. A positive test result was indicated by the appearance of both the control and test lines, while a negative result showed only the control line. All lateral flow assays were performed in triplicate.

### 2.11 Specificity and sensitivity of the RPA/PCR-CRISPR/Cas12a assay

To test the specificity of the one-pot RPA/PCR CRISPR/Cas12a assays, a total of 33 *P. sojae* isolates from different geographic origins, 37 isolates from 17 different oomycetes species, and 16 other fungi and bacteria were used (Table 1). To determine the sensitivity of the CRISPR/Cas12a assay, 10-fold serial dilutions of *P. sojae* purified gDNA ranging from 100 to 0.001 ng μL^−1^ were used as DNA templates. Nuclease-free H_2_O was used as the negative control. All assays were repeated in triplicate for each concentration of the gDNA template.

### 2.12 Feasibility of CRISPR/Cas12a detection using inoculated soybean tissues and collected field samples

To assess the practical application of the one-pot CRISPR/Cas12a detection for *P. sojae*, soybean seedlings were inoculated with *P. sojae* as described previously (Zhao et al., 2015). The DNA was extracted and evaluated using the developed RPA/PCR-CRISPR/Cas12a assays. Each experiment was repeated three times. In addition, a total of 20 soybean plants collected from different fields in Fujian and Hainan provinces were assayed using the RPA/PCR-CRISPR/Cas12a assays. The DNA was extracted from infected soybeans as described previously (Chen et al., 2013), and the RPA/PCR-CRISPR/Cas12a assay was performed as described above. Purified DNA of *P. sojae* was used as the positive control and healthy soybeans were used as negative control. The *P. sojae* infection was confirmed using the conventional isolation culture method for the identification of the pathogens based on morphological characteristics. Each experiment was repeated three times.

## 3 Results

### 3.1 The principle of the Cas-OPRAD assay

A schematic description of the Cas-OPRAD assay and the approximate time of each step are shown in Figure 1. The shortest time of Cas-OPRAD assay can reach about 0.5–1 h, including three primary steps: sample preparation, Cas-OPRAD assay, and visual readout. In the first step, the sample was prepared by using a rapid nucleic acids extraction method, and then the RPA or PCR technique ensured the efficient production of DNA amplification from the sample. In the first step, the sample was prepared by using a rapid nucleic acids extraction kit, the RPA or PCR technique was employed to enrich the target DNA fragments, which ensures the efficient production of DNA amplification from the sample. We utilized the principle of liquid surface tension and spatial isolation to separate the RPA/PCR reaction from sgRNA-Cas12 in a one-pot reaction. Specifically, the sgRNA-Cas12 complex was adsorption suspended on the top cover of the reaction tube, while the RPA or PCR reaction was located at the bottom of the tube. After the nucleic amplification reaction was completed, a simple centrifugation or manual shaking was used to mix the two components for reaction, this design avoids cross-contamination of various components in complex systems. For amplification product identification, the gRNA was specially designed to target RPA/PCR products, and the amplification products were recognized and cleaved, especially by the sgRNA and Cas12a complex. Furthermore, the Cas12a-sgRNA-target ternary complex activated the trans-cleavage activity of Cas12a, resulting in ultrafast cleavage of the ssDNA fluorescent probe and consequent fluorescent signal generation. The fluorescent signal generated from trans-cleavage was monitored by a fluorescence detector or LFS device.

### 3.2 Identification of specific sequence and specificity assay of Cas-OPRAD

The specificity of Cas OPRAD was carried out in three steps. Firstly, we determine sequence specificity through comparative genomic analysis, 1,187 candidate genes were identified from *P. sojae*. Pair-wise gene sequence alignments were then performed, and three *P. sojaespecific* genes were selected as the detection target candidates: PHYSODRAFT_299276, PHYSODRAFT_255386, and PHYSODRAFT_531894. Then, the primers were designed for these genes, and a conventional PCR assay was used to evaluate the specificity of the primer pairs. Primers So-F/So-R (Supplementary Table S1), designed from PHYSODRAFT_299276, successfully distinguished *P. sojae* from other plant pathogens by amplifying a 1,310 bp target band. In contrast, PHYSODRAFT_255386 and PHYSODRAFT_531894 amplified non-target bands from the gDNA of *P. sojae* (data not shown). These results suggest that the primers designed from PHYSODRAFT_299276 had good specificity for *P. sojae* detection. Therefore, a probe for PHYSODRAFT_299276 was used as the detection target.

### 3.3 Feasibility analysis, optimization for one-pot, and naked-eye detection

To verify the feasibility of our scheme, we first optimized the concentration of crRNA and the ratio of Cas12a to crRNA in the CRISPR/Cas12a assay, as the crRNA concentration plays a crucial role in CRISPR/Cas12a reaction. The optimal concentration of crRNA and the optimal ratio of Cas12a to crRNAs were determined to be 200 nM and 1:1, respectively (Supplementary Figure S1A). Under the optimized conditions of crRNA concentration and the ratio of Cas12a to crRNAs, we further optimized CRISPR/Cas12a reaction time and temperature. The ideal conditions for the cleavage were determined to be 38°C for 10 min (Supplementary Figures S1B,C).

**Figure 2:**
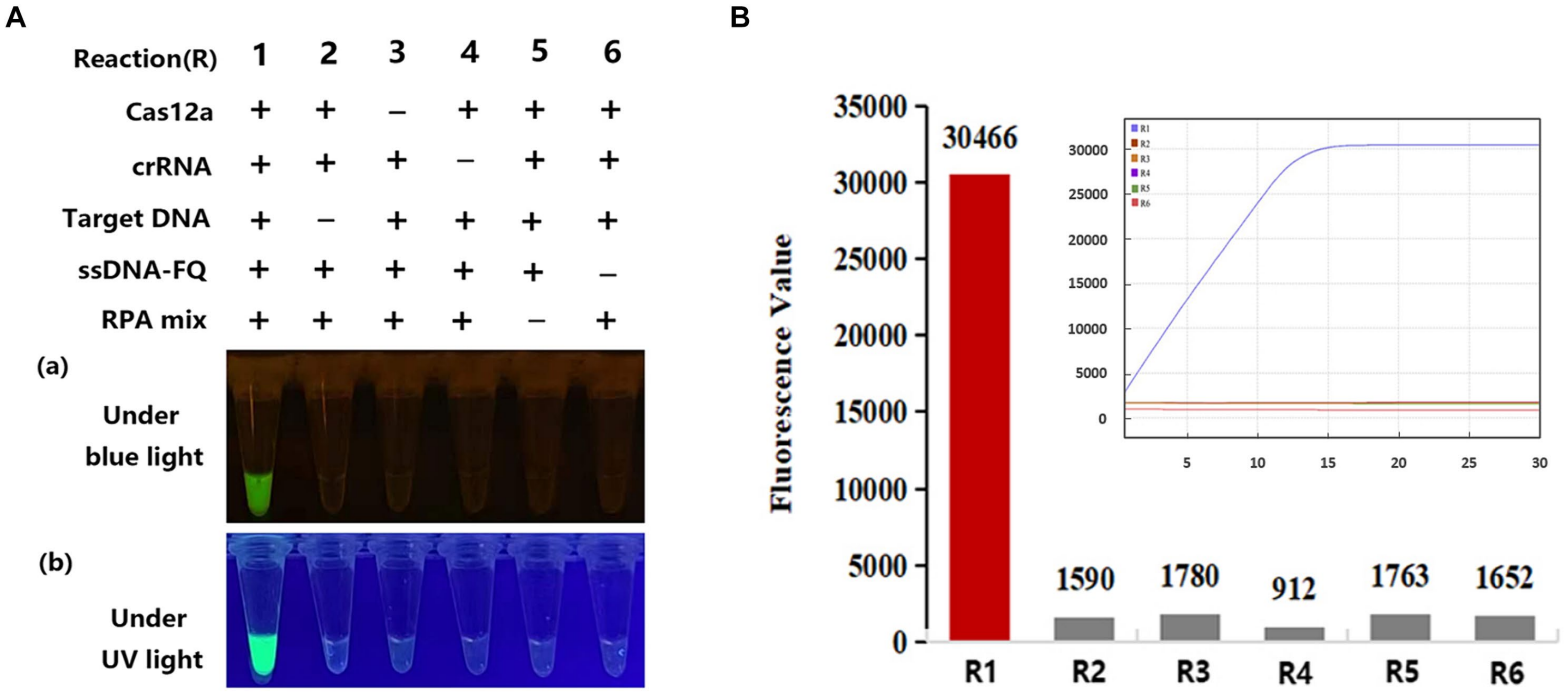
Evaluation of Cas-OPRAD reactions with various components. **(A)** RPA-CRISPR/Cas12a reaction. (a) Visualization under blue light; (b) visualization under UV light; **(B)** Fluorescent readout with quantitative PCR instrument. Each experiment was repeated three times with similar results.

To maintain the activity of Cas12a in one pot, according previous report by Saisawang et al. (2023), we adjusted the temperature of the heat cap in the PCR-CRISPR/Cas12a reaction to 50°C and trehalose was added into CRISPR/Cas reaction to help enzyme stabilization in target cleavage. The optimized concentration of trehalose for PCR-CRISPR/Cas12a was 0.6 mM (Supplementary Figure S1D). Therefore, subsequent PCR-CRISPR/Cas12a assays were carried out under these conditions. We tested eight reaction systems (reactions # 1–8) for PCR-CRISPR/Cas12a (Supplementary Figure S2) and six reactions (reactions # 1–6) for RPA-CRISPR/Cas12a, respectively (Figure 2). The generation of fluorescence comes from Cas12 cleavage FQ ssDNA probe molecules, after incubation at 38°C for 15 min and 5 ~ 10 min of CRISPR/Cas detection, only reaction #1, containing Cas12a, crRNAs, target DNA, ssDNA-FQ, and RPA or PCR reaction mixture, generated a naked-eye visual result that was visible under blue or UV light (Figure 2; Supplementary Figure S2). To further confirm the specificity and reaction time of the fluorescence signal, the one-pot RPA-CRISPR/Cas12a assay was analyzed on an AriaMx quantitative PCR instrument. These fluorescence value results demonstrate that the CRISPR/Cas12a assay provides a high fluorescence intensity value (>30,000 AU) within 15 min, which indicates that Cas-OPRAD can be offered a rapid, specific, and simple method for detecting *P. sojae* (Figure 2B).

### 3.4 Specificity and sensitivity of the Cas-OPRAD assay

**Figure 3:**
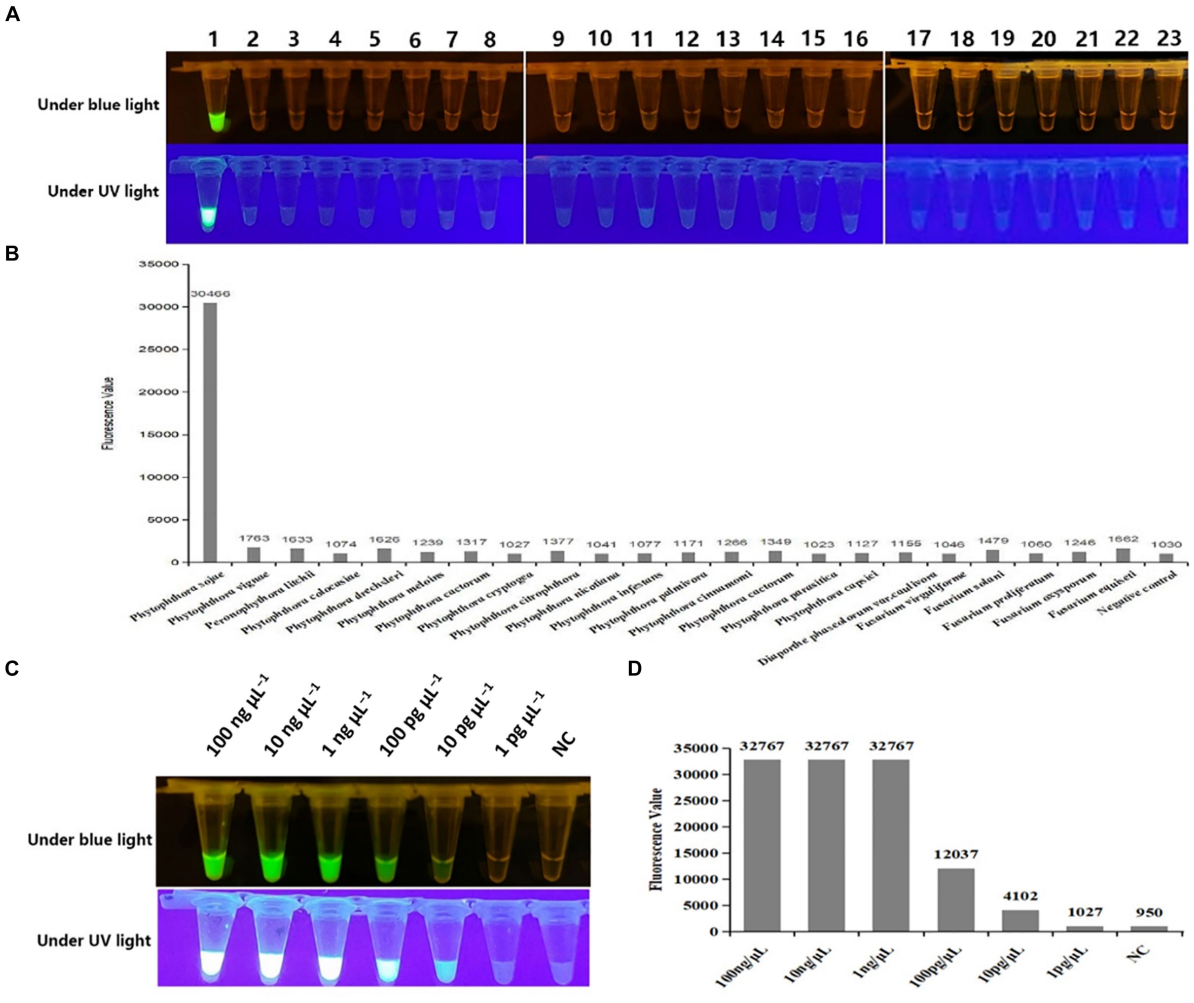
Specificity and sensitivity test of the Cas-OPRAD assay for detection of *P. sojae*. **(A)** Specificity visualization assay under blue and UV light; **(B)** Fluorescent readout detection for specificity assay of *P. sojae*; Lane 1: *Phytophthora sojae*; 2: *Phytophthora vignae*; 3: *Phytophthora infestans*; 4: *Phytophthora meloins*; 5: *Phytophthora cryptogea*; 6: *Phytophthora parasitica*; 7: *Phytophthora drechsleri*; 8: *Phytophthora capsici*; 9: *Phytophthora cactorum*; 10: *Phytophthora cinnamomi*; 11: *Phytophthora citrophthora*; 12: *Phytophthora colocasiae*; 13: *Phytophthora cactorum*; 14: *Phytophthora nicotiana*; 15: *Peronophythora litchii*; 16: *Phytophthora palmivora*; 17: *Diaporthe phaseolorum* var.*caulivora*; 18: *Fusarium virguliforme*; 19: *Fusarium equiseti*; 20: *Fusarium solani*; 21: *Fusarium proliferatum*; 22: *Fusarium oxysporum*; 23: Negative control. **(C)** Sensitivity visualization assay under blue and UV light; **(D)** Fluorescent readout detection of *P. sojae*.

To validate the specificity of Cas OPRAD detection, DNA templates were extracted from 33 *P. sojae* isolates and 53 isolates of other non-*P. sojae* were evaluated (Table 1). After the Cas-OPRAD reaction, only *P. sojae* showed a positive reaction, while no color change was observed for the other pathogens, including other *Phytophthora* spp. and other fungal and bacteria isolates from soybeans (Figures 3A,B; Supplementary Figures S3A,B). Notably, there was no cross-reaction with closely related species, such as *P. melonis*, *P. cinnamomi*, and *P. vignae*, which belong to the same clad 7b as *P. sojae*. These results demonstrate that the Cas-OPRAD assay developed in this study is highly specific for *P. sojae*.

To further evaluate the sensitivity of the CRISPR/Cas12a assay, 10-fold serially diluted DNA templates of *P. sojae* were used. As shown in Supplementary Figures S4A,B the detection limit for the PCR-CRISPR/Cas12a was 100 pg. μL^−1^, while the sensitivity of the RPA-CRISPR/Cas12a assay reached 10 pg. μL^−1^ in both fluorescence detection (Figures 3C,D). Notably, the sensitivity for Cas-OPRAD was found to be at least 10 times higher than that of the PCR-CRISPR/Cas12a method. These results suggest that the RPA-CRISPR/Cas12a-based assay is more sensitive for early detection of the soybean pathogen *P. sojae*.

### 3.5 Development of lateral flow strips platform for *Phytophthora sojae* detection

**Figure 4:**
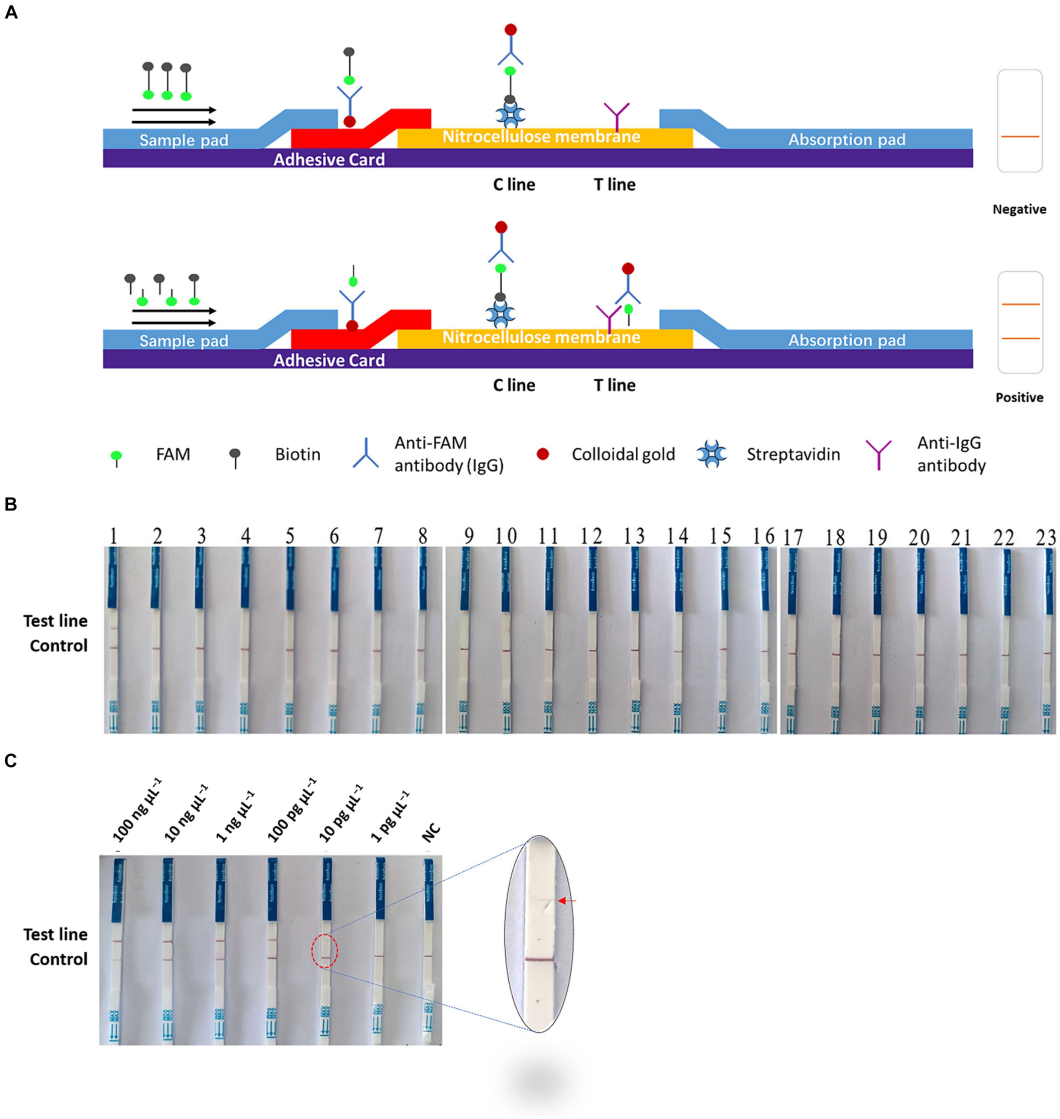
On-site Cas-OPRAD assays for detection of *P. sojae*. **(A)** Structure and design of the LFS. **(B)** Lateral-flow readout detection of *P. sojae*. Lane 1: *Phytophthora sojae*; 2: *Phytophthora vignae*; 3: *Phytophthora infestans*; 4: *Phytophthora meloins*; 5: *Phytophthora cryptogea*; 6: *Phytophthora parasitica*; 7: *Phytophthora drechsleri*; 8: *Phytophthora capsici*; 9: *Phytophthora cactorum*; 10: *Phytophthora cinnamomi*; 11: *Phytophthora citrophthora*; 12: *Phytophthora colocasiae*; 13: *Phytophthora cactorum*; 14: *Phytophthora nicotiana*; 15: *Peronophythora litchii*; 16: *Phytophthora palmivora*; 17: *Diaporthe phaseolorum. **(C)** The sensitivity assay of lateral flow strips readout detection for P. sojae. Lane 1:100 ng μL−1; 2: 10 ng μL−1; 3: 1 ng μL−1; 4: 100 pg μL−1; 5: 10 pg μL−1; 6: 1 pg μL−1; and 7: NC (negative control).*

The Cas-OPRAD assay is performed at an isothermal temperature, making it potentially suitable for on-site detection. In addition to fluorescence detection, we also utilized lateral flow strips (LFS) for a visual CRISPR/Cas12a assay that can facilitate on-site detection of *P. sojae*. The principle of the test strip is shown in Figure 4A, the structure of LFS incorporated an absorbent pad, a conjugate pad, a nitrocellulose (NC) membrane, and a sample pad on a plastic adhesive backing card. The capture reagents streptavidin and anti-IgG antibody were dispensed onto the NC membrane, and each band was separated by 5 mm. When there is no target in the system, the conjugate was captured by the streptavidin through biotin, the control line became red because of the aggregation of the colloidal gold in this area, while the test line without the flow-through of the conjugate had no color change. However, if there are targets in the system, the ssDNA reporter is cleavaged by CRISPR/Cas12a, and FAM and biotin molecules are separated. After the integration of ssDNA-biotin captured by streptavidin at the C line, the remaining conjugate of FAM-ssDNA and anti-FAM antibody would flow into the test line and be captured by anti-lgG antibody. As expected, bands appeared on both the test line and control line of the strip in a positive reaction, while only a band appeared on the control line in a negative reaction. Therefore, the lateral flow assay format provides a rapid, simple, instrument-free, on-site field detection method for *P. sojae*.

The specificity and sensitivity of the Cas-OPRAD LFS platform were also evaluated for the detection of *P. sojae*. The specificity test results were visualized using LFS tests (Figure 4B). In the LF-OPRAD assay, red bands at the T line were only observed for the Cas-OPRAD products of the *P. sojae* DNA sample, and no red band at the T line was observed for reactions with other pathogens. These results indicate that the developed Cas-OPRAD platform for the *P. sojae* pathogens was highly specific. In sensitivity tests, the results from LFS indicated are same sensitivity as for the Cas-OPRAD assays. For example, a red band at the T line was observed for reactions at all *P. sojae* DNA dilutions except for 1 pg. μL^−1^ and NC (Figure 4C), indicating that the sensitivity of Cas-OPRAD was 10 pg. μL^−1^, these results indicate that the on-site platform could be used for *P. sojae* pathogen detection without dedicated professional equipment.

### 3.6 On-site detection using inoculated soybeans and field soybean samples

**Figure 5:**
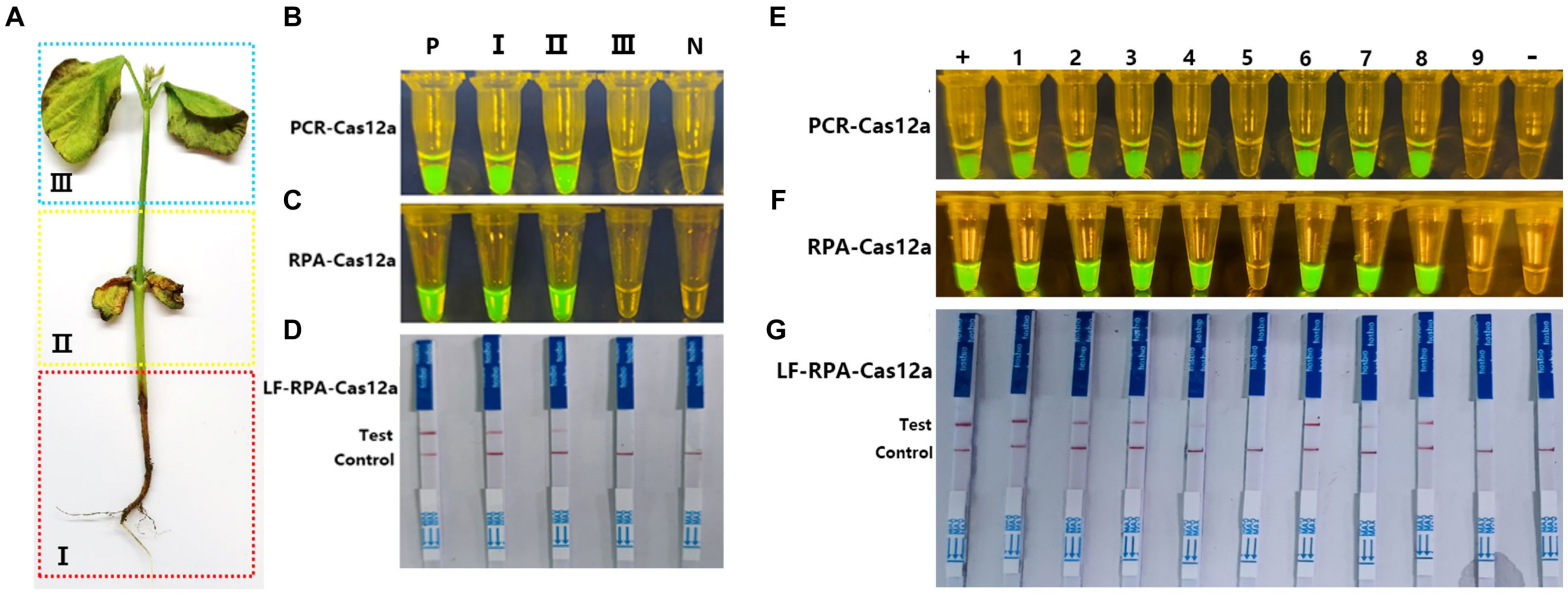
Application of Cas-OPRAD assays for detection of *P. sojae* in filed samples. **(A)** Artificial division of different areas of the soybean represents the varying stages of infection; **(B)** Blue and UV light visualization assay of one-pot PCR-Cas12a in inoculated soybean seedlings; **(C)** Blue and UV light visualization assay of one-pot RPA-Cas12a in inoculated soybean seedlings; **(D)** One-pot RPA-Cas12a LFS assay in filed soybean sample; +: Positive control; −: negative control; Lane1: Region I; Lane 2: Region II; Lane3: Region III. **(E)** one-pot PCR-Cas12a LFS assay; **(F)** one-pot RPA-Cas12a LFS assay; **(G)** RPA-Cas12a LFS assay in filed soybean sample. +, Positive control; −, negative control; Lane1-8: naturally infected soybean root samples; 9: healthy soybean sample.

To verify the validity and feasibility of the Cas-OPRAD for diagnosing soybean samples, the DNA was extracted from three regions (I: roots and underground stems. II: cotyledons and stem above the part of ground. III: epicotyl and first leaf) of inoculated soybean seedlings (Figure 5A). As shown in Figures 5B–D, positive reactions were observed in regions I and II, but not in region III or healthy soybeans using Cas-OPARD or PCR-CRISPR/Cas12a, the result indicates that under artificial inoculation, the *P. sojae* infection in the roots of soybeans is higher than that in the leaves. Therefore, in field sample collection, it is necessary to prioritize collecting regions I or II for Cas-OPARD analysis.

According to the results above, a total of 20 field soybean samples collected from natural fields of Fujian and Haina province, China, were used for Cas-OPARD assay or PCR-CRISPR/Cas12a with both fluorescence and lateral flow readouts. Of the 20 collected samples, 16 were positive and four were negative using both the Cas-OPARD, the results obtained by using the PCR-CRISPR/Cas12a method are consistent with those obtained by Cas-OPRAD (Figures 5E,F). The detection results showed 100% consistency between the visual detection under blue or UV light and the lateral flow strip readout (Figures 5F,G). These results demonstrate the high detection capabilities of the CRISPR/Cas12a assay for *P. sojae*.

## 4 Discussion

The identification of a specific target gene is critical for the development of accurate and sensitive molecular diagnosis methods. Although several genes, such as the internal transcribed spacer (ITS) region, β-tubulin (TUB) loci, elongation factor 1α, and ras-related protein *Ypt1* (YPT), have been used to design primers for detecting *Phytophthora* (Kroon et al., 2004; Blair et al., 2008). However, molecular detection based on these target genes may pose false positives due to high homology (Kunadiya et al., 2017). Therefore, the combination of comparative genomics and bioinformatics has been applied to identify specific target genes for various plant pathogen species, including *Pectobacterium carotovorum* (Ahmed et al., 2018), *P. infestans* (Kong et al., 2020), *P. cinnamomi* (Chen et al., 2022), *Magnaporthe oryzae Triticum* (MoT) (Kang et al., 2021), and *Fusarium circinatum* (Xu et al., 2023). In this study, we performed a comparative genomic analysis based on public genomic sequence data of several oomycetes species to identify a candidate detection target gene that is specific to *P. sojae*. We identified a single-copy gene (PHYSODRAFT_299276) and developed RPA/PCR CRISPR/Cas12 assays to verify its specificity using 33 *P. sojae* isolates and 53 isolates of other non-*P. sojae* species. Moreover, this assay did not show cross-reaction with closely related species clade 7b, *P. melonis*, *P. cinnamomi*, and *P. vignae.* RPA/PCR CRISPR/Cas12 assay that showed 100% inclusivity and 100% exclusivity when tested among closely related species. Our results suggest that this assay is highly specific for *P. sojae* detection.

One-pot CRISPR/Cas coupled with nucleic acid amplification detection streamlines genetic analysis by integrating multiple steps into a single reaction. This synergy enhances efficiency, reduces time, and minimizes contamination risks, crucial for applications like pathogen detection, genetic screening, and diagnostics. By combining CRISPR’s precision gene detection with amplification’s sensitivity, it enables rapid and accurate identification of target sequences, revolutionizing research, and medical diagnostics (Wang R et al., 2021; Hu et al., 2022). The simplicity and versatility of this approach hold immense potential for advancing various fields, from healthcare to agriculture and beyond, promising faster, more accessible, and reliable genetic analysis. To reduce the risks of cross-contamination and improve the convenience of on-site detection of *P. sojae*, we developed a one-pot CRISPR/Cas12a OPRAD assay. In the One-pot design, we utilized the principle of liquid surface tension and spatial isolation to separate the RPA/PCR reaction from the sgRNA-Cas12 a one-pot reaction. This integrated strategy initially provided a new idea for on-site diagnosis and epidemic control of COVID-19 (Ding et al., 2020). Additionally, to maintain the activity of Cas12a in one pot PCR-CRISPR/Cas12, we adjusted the temperature to 50°C of the heat cap and optimized 0.6 mM trehalose to maintain the stability in one-pot PCR-CRISPR/Cas12a reaction. As a nonreducing disaccharide, trehalose can provide proteins with the most stability in a one-pot reaction, as they form hydrogen bonds with the protein to minimize damage from desorbing the hydration shell (Lin et al., 2022; Ghouneimy et al., 2023). CRISPR/Cas12a reagents were pre-loaded into the inner wall of the tube lid. Following RPA/PCR amplification, the reagents were mixed into the reaction solution by centrifugation, avoiding potential aerosol contamination from re-opening the tube lid post-amplification. This assay minimizes potential cross-contamination issues by avoiding the need to reopen the reaction tube lid after the RPA reaction.

Isothermal amplification techniques have emerged as powerful alternatives to PCR (Polymerase Chain Reaction) due to their simplicity, rapidity, and applicability in various settings. While PCR relies on thermal cycling to amplify DNA, isothermal amplification methods operate at a constant temperature, making them more accessible for point-of-care diagnostics and field applications (Qi et al., 2018). RPA employs recombinase proteins to facilitate strand exchange, enabling DNA amplification at a constant temperature (usually 37–42°C). RPA reactions are highly specific and can be completed within 20–30 min, making them suitable for rapid diagnostics in resource-limited settings (Piepenburg et al., 2006; Guo et al., 2023). These isothermal techniques offer several advantages over PCR, including faster turnaround times, simplified instrumentation requirements, and tolerance to inhibitors present in crude samples. They have been extensively applied in fields such as infectious disease diagnosis, food safety monitoring, and environmental surveillance. As isothermal amplification methods continue to evolve, they hold immense promise for decentralized molecular testing and point-of-care applications (Sánchez et al., 2022; Kasfy et al., 2024). Although our results show that both amplification methods can be combined with one-pot CRISPR/Cas12 detection, however, the one-pot RPA shows higher sensitivity (about 10 times) since RPA has a higher amplification efficiency in the one-pot-CRISPR/Cas12 reaction, indicating that Cas-OPRAD based on RPA-CRISPR/Cas is more practical for detection due to lower reaction temperature, shorter detection time, and is easier to operate in the field. Moreover, the amplification temperature of the RPA reaction allows it to be paired with CRISPR/Cas12a cleavage in a one-pot reaction. This approach simplifies the assay by using a single temperature and a single tube for the amplification reaction, making it amenable to on-site detection without the need for highly equipped laboratories or well-trained personnel.

On-site detection is pivotal for swift response, cost-efficiency, and preventive action in various fields. It enables immediate identification of substances, pathogens, and anomalies, saving time and resources by eliminating the need for off-site analysis (Hu et al., 2017; Bai et al., 2019; Zhu et al., 2022). The established Cas-OPRAD has powerful field sample processing capabilities, as illustrated in Scheme 1. The one-pot CRISPR/Cas12a-based detection platform works with four processes that integrate (i) Sample preparation (ii) PCR or RPA amplification of the target DNA, sequence-specific recognition, and trans-cleavage by Cas12a/crRNA (Cas-OPRAD assay), and (iii) visual readout of results. This streamlined approach provides a powerful tool for on-site detection of this destructive soybean pathogen, which can straightforward visual interpretation of results by the naked eye without requiring specialized equipment.

## 5 Conclusion

In this study, we developed a one-pot RPA/PCR-CRISPR/Cas12a detection platform for sensitive and specific detection of the soybean pathogenic oomycete *P. sojae*, named Cas-OPRAD. The advantages of Cas-OPRAD are shown in Table 2 compared with previously reported methods. The Cas-OPRAD assay has the advantages of sensitivity (<10 pg./μL), due to the RPA amplification and trans cleavage of activated Cas12a protein. The assay demonstrates exceptional analytical specificity without cross-reactivity against closely related species with specific RPA primers and CRISPR sgRNA design. In addition, to achieve on-site detection, we utilized the principle of liquid surface tension and spatial isolation to separate the RPA/PCR reaction from sgRNA-Cas12 in a one-pot reaction. We provide a straightforward visual readout for the soybean pathogen *P. sojae* via both fluorescent and lateral flow detection modalities, which extends the application of Cas-OPRAD to point-of-care self-diagnoses. The whole test procedure can be performed in the field with simple operator training, using a smart thermos cup (Zhu et al., 2022), and visual inspection using a LFS device. In field sample tests, the time required for the whole process can be shortened to 40 min for all 20 field soybean samples, considerably shorter than conventional PCR or RPA-CRISPR/Cas assay. By coupling target amplification by RPA with Cas12a-mediated cleavage in a streamlined single-reaction format, this platform enables on-site application for early and precise identification of *P. sojae* outbreaks to empower effective disease management. Overall, this integrated isothermal CRISPR-based diagnostic tool has tremendous potential to aid in monitoring and control of destructive soybean diseases attributed to *P. sojae* infection.

**Table 2:** Comparison of performance between recent detection techniques and Cas-OPRAD.

Platform	Steps	Specificity	Sensitivity	Test time	One-pot	On-site	References
qPCR and RPA	(1) qPCR (2) RPA	100%	qPCR (100 fg) qPCR (10 pg)	>60 min	Yes	No	Rojas et al. (2017)
RPA-LFD	(1) RPA (2) LFS visual assay	55.4%	10 pg./50 μL	>30 min	No	No	Dai T. et al. (2019)
RPA-CRISPR/Cas12a	(1) RPA (2) CRISPR/Cas Fluorescence assay	100%	10 pg./μL	>30 min	No	No	Guo et al. (2023)
RPA-CRISPR/Cas12a	(1) RPA (2) CRISPR/Cas Fluorescence assay (3) LFS visual assay	100%	14.5–24.6 copies/μL	>60 min	No	Yes	Sun et al. (2024)
Cas-PfLAMP	(1) LAMP or RT-LAMP (2) CRISPR/Cas Fluorescence assay (3) LFS visual assay	100% Compare PCR	3–9 copies (<10 copies)	~ 50 min	No	Yes	Zhu et al. (2022)
Bio-SCAN	(1) RPA (2) CRISPR/Cas Fluorescence assay (3) LFS visual assay	100%	alleles assay	<60 min	No	Yes	Sánchez et al. (2022)
NALFIA	(1) RPA (2) CRISPR/Cas Fluorescence assay (3) LFS visual assay	1,000% Compare PCR	0.001 μg/μL	~25 min	No	Yes	Kang et al. (2021)
Cas-OPRAD	(1) RPA (2) CRISPR/Cas Fluorescence assay (3) LFS visual assay	100%	10 pg./μL	~30 min	Yes	Yes	This work

## Data availability statement

The original contributions presented in the study are included in the article/Supplementary material, further inquiries can be directed to the corresponding authors.

## Author contributions

ZL: Writing – review & editing, Writing – original draft, Validation, Methodology, Formal analysis, Data curation. WF: Writing – review & editing, Writing – original draft, Validation, Methodology, Formal analysis, Data curation. ZZ: Writing – review & editing, Validation, Methodology, Formal analysis, Data curation. SL: Writing – review & editing, Methodology, Data curation. ML: Writing – review & editing, Methodology, Data curation. JD: Writing – review & editing, Methodology, Data curation. ZW: Writing – review & editing, Methodology, Data curation. FL: Writing – review & editing, Supervision, Investigation, Funding acquisition, Data curation, Conceptualization. QC: Writing – review & editing, Writing – original draft, Supervision, Project administration, Investigation, Funding acquisition, Conceptualization.

## Footnotes

^1^ https://genome.jgi.doe.gov/Physo3/Physo3.home.html

^2^ http://primerexplorer.jp/e/

^3^ http://grna.ctegd.uga.edu
