# Supplementary material for: Cas-OPRAD: a one-pot RPA/PCR CRISPR/Cas12 assay for on-site Phytophthora root rot detection
Source: Front Microbiol. 2024 Jun 5;15:1390422. doi: 10.3389/fmicb.2024.1390422 (PMC11188302; doi:10.3389/fmicb.2024.1390422)
Supplement: Supplementary file 1 [file Data_Sheet_1.docx]

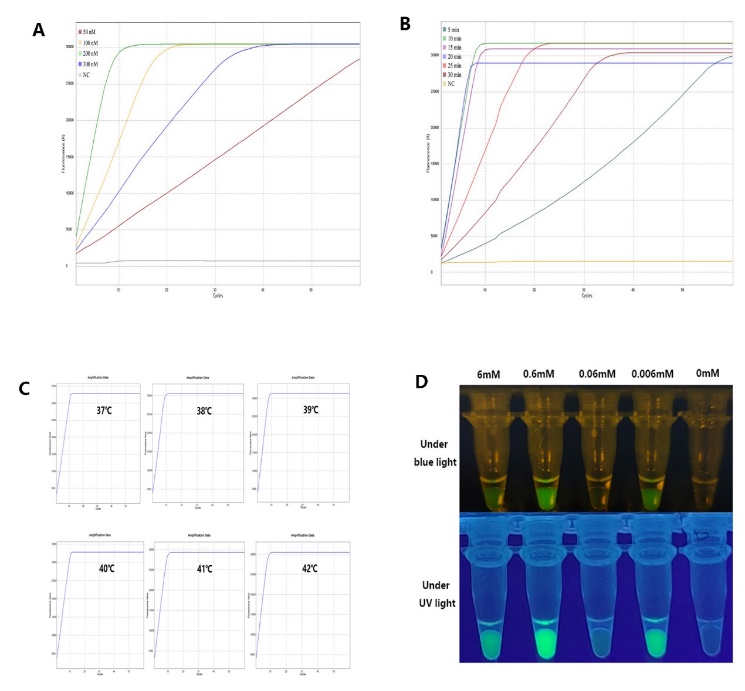


**Figure S1. Optimization of CRISPR/Cas12a assay.** Optimization of CRISPR/Cas12a results under different reaction conditions by (A) different concentrations of Cas12a and sgRNA in the case of Cas12a: sgRNA=1:1; (B) different time of RPA reaction; (C) different temperatures of RPA; (D) concentration of trehalose for PCR-CRISPR/Cas12a. M, DL2000 DNA marker. N, negative control.

**Figure S2. Evaluation of one-pot PCR-CRISPR/Cas12a reactions with various components.** (A) PCR-CRISPR/Cas12a reaction; (a) Visualization under blue light; (b) Visualization under UV light.


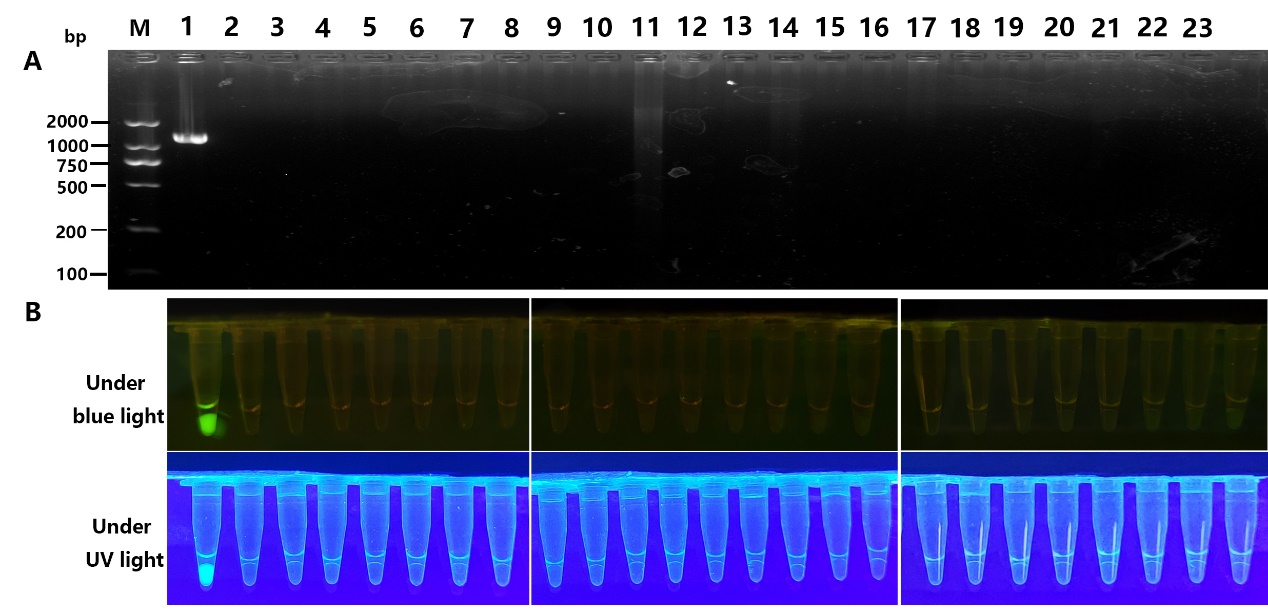


**Figure S3. Specificity test of the one-pot PCR-CRISPR/Cas12a assay for detection of *P. sojae*.** (A) PCR amplification with primers So-F/So-R; (B) PCR-CRISPR/Cas12a visualization under blue and UV light. Lane M: Marker; Lane 1: *Phytophthora sojae*; 2: *Phytophthora vignae*; 3: *Phytophthora infestans*; 4: *Phytophthora meloins*; 5: *Phytophthora cryptogea*; 6: *Phytophthora parasitica*; 7: *Phytophthora drechsleri*; 8: *Phytophthora capsici*; 9: *Phytophthora cactorum*; 10: *Phytophthora cinnamomi*; 11: *Phytophthora citrophthora*; 12: *Phytophthora colocasiae*; 13: *Phytophthora cactorum*; 14: *Phytophthora nicotiana*; 15: *Peronophythora litchii*; 16: *Phytophthora palmivora*; 17: *Diaporthe phaseolorum* var.*caulivora*; 18: *Fusarium virguliforme*; 19: *Fusarium equiseti*; 20: *Fusarium solani*; 21: *Fusarium proliferatum*; 22: *Fusarium oxysporum*; 23: Negative control.


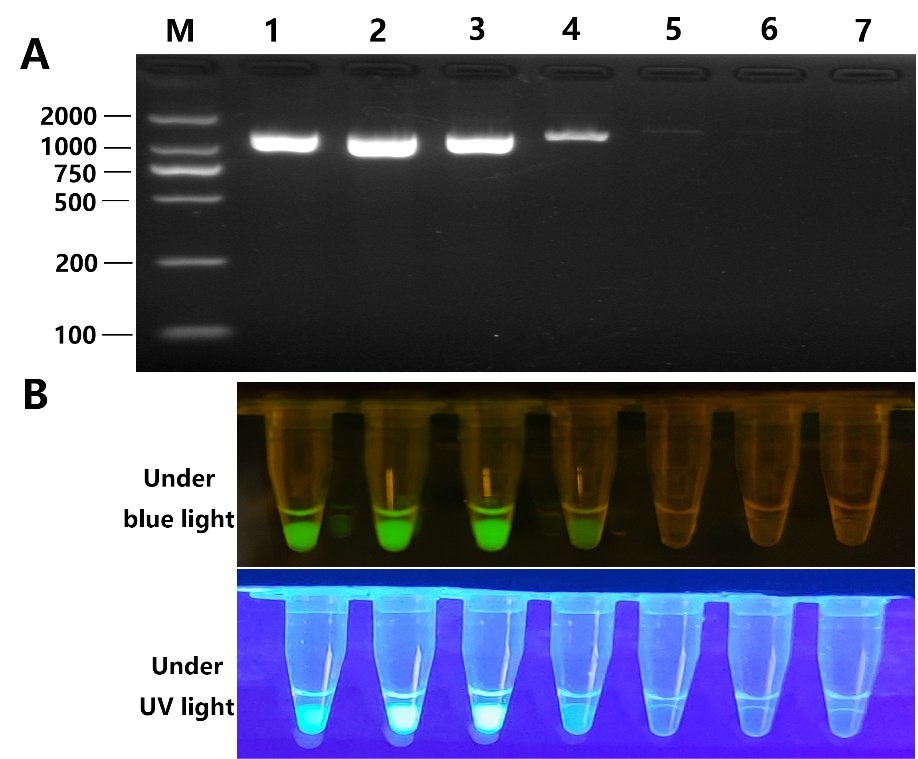


**Figure S4. Sensitivity test of the one-pot PCR-CRISPR/Cas12a assay for detection of *P. sojae*.** (A) PCR amplification with primers So-F/So-R; (B) RPA and visualization under blue and UV light. Lane M, Marker; Lane 1:100 ng μL^−1^; 2: 10 ng μL^−1^; 3: 1 ng μL^−1^; 4: 100 pg μL^−1^; 5: 10 pg μL^−1^; 6: 1 pg μL^−1^; and 7: NC (negative control).

Table S1. list of primers, sgRNA, and probes used in this study

| Primers or Probes | Sequences（5’ → 3’） | Reference |
| --- | --- | --- |
| So-F | CACTTGTGGCTCTGTAG | For PCR |
| So-R | GCAGCTTTCTCTAGGTAG | For PCR |
| SoQ-F | TGGTAGTGCAGTCTCTATC | For real-time PCR |
| SoQ-R | TGATCCAACCTCATTGAC | For real-time PCR |
| SoQ | FAM-CATGTTGCCACCTGGATTCGA-BHQ1 | For real-time PCR |
| RPA-F | CGCCATACGTTTTCCACTACGGATAAAGAG | For RPA |
| RPA-R | AATTGCTTGATCCAACCTCATTGACGCAGC | For RPA |
| sgRNA | UAAUUUCUACUAAGUGUAGAUGUCAUCUCGGUGAACGACACCUU | For Cas12a |
| FQ-reporter 1 | FAM-TTATT-BHQ1 |  |
| FQ-reporter 2 | FAM-TTATT-Biotein |  |
